# Effect of date palm (*Phoenix dactylifera* L.) leaves on productive performance of growing lambs

**DOI:** 10.1007/s11250-020-02493-2

**Published:** 2021-01-05

**Authors:** A. A. Mahrous, A. A. H. El-Tahan, Y. H. Hafez, M. A. El-Shora, O. A. Olafadehan, H. Hamdon

**Affiliations:** 1 Animal Production Research Institute, Agricultural Research Center, Dokki, Giza, Egypt; 2 Department of Animal Science, University of Abuja, Abuja, P.M.B. 117 Nigeria; 3 Department of Animal Production, Faculty of Agriculture, New Valley University, New Valley, Egypt

**Keywords:** Additive, Date palm leaves, Silage, Digestibility, Weight gain

## Abstract

Eighteen 4-month-old lambs, with a mean live weight (LW) of 19.47 ± 0.20 kg, were used to evaluate the nutritive value of date palm leaves (DPL) ensiled with different additives in a completely randomized design. Lambs were stratified into three groups of 6 lambs each and fed a control diet comprising 60% concentrate feed mixture (CFM) and 40% DPL silage (T1). In other treatments, the DPL silage (DPLS) of the control treatment was replaced with EM_1_ additive-treated DPLS (T2) or El-Mofeed additive-treated DPLS (T3). Apparent digestibility, total digestible nutrient, digestible crude protein, dry matter intake, daily weight gain (DWG), price of DWG, daily profit, and economics of feed efficiency were higher (*P* < 0.05) for the additives-treated DPLS relative to the control, with T2 enhancing these parameters compared with T3. With exception of ruminal pH, which was reduced, concentrations of ruminal NH_3_-N and total volatile fatty acids (VFA) increased 4 h post feeding. However, ruminal NH_3_-N and total VFA were greater (*P* < 0.05) for the additives-treated DPLS, with T2 producing higher values than T3. Ruminal pH and feed cost/kg LW gain were lower for T2 relative to other treatments. Blood constituents were within the normal ranges for lambs, though slightly altered by treatments. Whereas serum total protein, albumin, and globulin were affected (*P* < 0.05) in this rank order, T1 < T3 < T2, other serum parameters were not affected. Relative feed cost and relative daily profit were lower and higher respectively for T2 than for T3. It is concluded that additives-treated DPLS is nutritionally superior to untreated DPLS as a roughage source in total mixed rations fed to growing lambs. However, for improved performance of the lambs and economic benefits, EM_1_-treated DPLS is recommended.

## Introduction

Date fruit production yields several crop residues, including date palm fronds or leaves (DPL), petioles, racemes (without the dates), and pedicels. These by-products are usually used as feed for animals during winter, though they can be used year round (Genin et al. 2004). A date palm tree produces 13.5–20 kg DPL annually (Chehma and Longo 2001). FAO (2011) reported that 1.15 million ha of land were planted with palm trees in Egypt. Base on average density of 100–125 date palm trees/ha, it was estimated that 1.9–2.4 million tons of dry leaves or fronds of date palm were available annually (Genin et al. 2004). Though DPL is available in large amounts, its utilization as animals feed is very limited due to the low nutritive value (Khalifa 2019). The composition of the leaves is 54.12% dry matter (DM), 89.86% organic matter (OM), 8.51% crude protein (CP), 28.48% crude fibre (CF), 59.11% neutral detergent fibre (NDF), 42.87% acid detergent fibre (ADF), 24.69% hemicellulose, and 16.24% lignin (Olafadehan and Adebayo 2016). Due to anaerobic fermentation during silage preparation, silage has been used to improve the nutritive value of poor-quality roughages (Olafadehan and Adebayo 2016; Olafadehan and Okoye 2017). Date palm leaves have been reported to possess good silage quality when mixed with additives such as wheat bran and urea (Khorchani et al. 2004). Arbouche and Arbouche (2008) reported improved CP content and DM digestibility of DPL treated with ammonia. Although previous studies demonstrated the potentials of DPL in the diets of lactating cows (Bahman et al. 1997) and growing rabbits (El-Bordeny and Abdel-Azeem 2007), biological (use of desirable organisms for fermentation) and chemical (use of nutritive materials) treatment of DPL could further enhance its nutritive quality in livestock diet. It is expected that ensiling of crop residues with desirable organisms or nutritive additive would affect the silage quality differently due to different modes of action. However, information on nutritive value of additives-treated DPL silage (DPLS) in the diets of livestock is limited. It was hypothesized that feeding additive-treated DPLS as a source of roughage in the diets of growing lambs would improve feed intake, nutrient digestibility, ruminal fermentation, and growth performance, without compromising the blood metabolic profile. The aim of study was to evaluate the feed value of additive-treated DPL in the diets for growing lambs.

## Materials and methods

### Additives for ensiling and silage preparation

Two different additives were used for treating DPL before ensiling. The first additive, EM_1_, is a product of EMRO Organization in Japan (EM_1_ Research Organization, Inc., Takamiyagi Bldg. 2F, 2-9-2 Gameko, Ginowan-shi Okinawa, Japan), containing a combination of 70 to 80 different types of beneficial microorganisms. The principal organisms are photosynthetic bacteria (phototrophic bacteria), lactic acid bacteria, yeasts, actinomycetes and fermenting fungi (Higa 1993). The second additive, El-Mofeed, is a nutritive product produced by El-Nobaria Station, Animal Production Research Institute, Agriculture Research Centre, Dokki, Giza, Egypt. It is made up of 91% molasses, 2.5% urea, and 6.5% solution containing a mixture of some mineral salts (calcium and manganese, magnesium, cobalt, copper, zinc, iodine, iron, potassium, and phosphorus) and vitamins A and D. Briefly, DPLS was produced by dissolving 5% each of the EM_1_ and El-Mofeed additives in 90 L of water. Each solution was mixed with 100 kg of DPL until the moisture content of the mixture was about 65–70%. The treated DPL was packaged into polyethylene bags, put in air-tight containers, and left for 3 weeks after which the containers were opened and the silages air-dried before feeding.

### Lambs, diets, and management

The study was carried out at Sakha Experimental Station, Animal Production Research Institute, Agriculture Research Centre, Egypt. Eighteen crossbred ram lambs (½ Finnish Landrace × ½ Rahmani) at 4 months of age (19.47 ± 0.20 kg LW) were used in a completely randomized design. They were weighed, divided into three homogenous groups (6 lambs/group), and housed individually in a 120-day feeding trial. Lambs were fed a control diet (T1) comprising 60% DM concentrate feed mixture (CFM) and 40% DM DPL to meet their nutrient requirements according to NRC (1985). In the other experimental diets, the 40% DPLS in the control diet was replaced with 40% EM_1_ additive-treated DPLS (T2) or 40% El-Mofeed additive-treated DPLS (T3). Animals were randomly assigned to one of three experimental treatments and fed ad libitum, twice daily at 08:00 h and 15:00 h, to provide allowance for orts collection. The CFM and DPLS were sampled daily, composited weekly, oven dried at 60 °C for 48 h, and stored pending chemical analyses. The chemical composition of component of the diets is shown in Table 1.

**Table 1:** Chemical composition (% DM unless otherwise stated) of untreated and treated date palm leaves silage and concentrate feed mixture (CFM)

Item	T1	T2	T3	CFM*
DM (% fresh matter)	94.44	90.50	89.30	88.7
OM	92.43	87.38	85.55	92.82
CP	4.20	7.50	5.60	14.16
CF	43.01	39.32	41.87	11.05
EE	2.31	1.15	1.16	2.3
NFC	42.91	39.41	36.92	65.31
NDF	81.65	74.20	77.32	27.79
ADF	67.72	59.31	61.22	8.86
ADL	37.84	26.05	28.30	2.89
Cellulose	29.88	33.26	32.92	5.88
Hemicellulose	13.93	14.89	16.10	18.89

*Concentrate feed mixture (CFM) consisted of 38% ground yellow corn, 22% undecorticated cotton seed meal, 7% soybean meal, 12% wheat bran, 13% rice bran, 5% cane molasses, 2% lime stone, and 1% common salt

*T1* untreated DPLS, *T2* DPLS treated with EM_1_, *T3* DPLS treated with El-Mofeed

### Feed intake and apparent digestibility

Feed intake was measured daily throughout the experimental period, while nutrient digestibility coefficients were determined using six animals for each ration at the end of the experimental period. Faeces were collected daily and oven dried at 60 °C for 48 h. The oven-dried daily faecal samples of each ram were bulked, thoroughly mixed, subsampled, and kept in a refrigerator for chemical analysis. Samples of feed, orts, and faeces were ground to pass a 1-mm screen using a Wiley mill (Arthur H. Thomas, Philadelphia, PA, USA) and analysed for dry matter (DM, method ID 934.01), ash (method ID 942.05), EE (method ID 956.02), and crude protein (CP, method ID 954.01) according to the methods of AOAC (2006). Fibre fractions, neutral detergent fibre (NDF), acid detergent fibre (ADF), and acid detergent lignin (ADL) were determined according to Van Soest et al. (1991). Hemicellulose and cellulose were calculated as the differences between NDF and ADF and ADF and ADL, respectively. Non-fibre carbohydrate NFC was estimated as NFC = 100 – (NDF + CP + EE + ash)%.

### Ruminal fluid collection and analysis

At the end of the experiment trials, rumen fluid samples were taken from six animals of each group using stomach tube before feeding and 4 h post feeding. The samples were filtered through 3 layers of gauze. Ruminal pH was measured immediately using pH metre (Orion Research, model 201/digital pH metre). Ammonia nitrogen (NH_3_-N) concentration was measured according to Conway and O'Mally (1957). Total volatile fatty acids (VFA) concentration was determined by the steam distillation method according to Abou-Akkada and Osman (1967).

### Blood collection and analysis

Blood samples were drawn from the jugular veins of six animals of each group at 4 h after morning feeding and centrifuged for 20 min at 3000 rpm. The supernatant was collected, frozen, and stored at − 20 °C for subsequent analysis. Blood serum was analysed for total protein (Armstrong and Carr 1964), albumin (Doumas et al. 1971), creatinine (Folin 1994), urea (Siest et al. 1981), and cholesterol (Fassati and Prenciple 1982). Amino aspartate transaminase (AST) and alanine transaminase (ALT) were determined calorimetrically by the methods of Reitman and Frankel (1957), using commercial kits (Biodiagnostic, Dokki, Giza, Egypt). Globulin concentration was calculated by subtracting albumin concentration from the corresponding total protein concentration.

### Statistical analysis

Data were analysed as a completely randomized design using the general linear model of SAS 9.1.3 (SAS Institute Inc., Cary, North Carolina, USA). The following mathematical model was used:

Y_ij_ = μ + T_i_ + e_ij_

where *Y*_*ij*_ is the observation on the experimental unit, *μ* is the overall mean, *T*_*i*_ is the effect due to treatment, and *e*_*ij*_ is the experimental error.

Duncan’s multiple range test was used to separate significant treatment means, and significance was declared if *P* < 0.05.

## Results and discussion

### Chemical composition

Additive treatments of DPL decreased the DM, OM, fibre fractions, and NFC but increased CP (Table 1). EM_1_ and El-Mofeed increased DPLS CP by 74.3 and 33.3% but decreased NDF, ADF, and ADL by 9.0 and 5.3%, 12.4 and 9.6%, and 31.2 and 23.2%, respectively. The chemical composition of the untreated DPLS is consistent with the earlier reports (Pascual et al. 2000; Kafilzadeh et al. 2009; El-Waziry et al. 2013). The increased CP and decreased fibre fractions indicate that additive treatments improved the nutritive value of DPLS. The results agree with those of Villas-Boas et al. (2002) who observed that biological treatment of poor-quality by-products increased their nutritional value because of significant increase in the concentrations of CP and NFC (soluble carbohydrates) and reduction in fibre fractions. The reduced fibre fractions of the additives-treated DPLS may be due to the catabolic action of certain fermentative microorganisms which utilize carbohydrate as a carbon source for growth and formation of microbial protein (Olafadehan et al. 2012; Olafadehan and Adebayo 2016). The relatively higher CP and lower fibre fractions of the T2 compared to T3 show that DPLS treated with EM_1_ (fermentative organisms containing additive) improved anaerobic fermentation efficiency and thus nutritive value of DPLS compared to DPLS treated with El-Mofeed (nutritive additive containing molasses, urea, some minerals, and vitamins A and D).

### Apparent digestibility and nutritive value

Additive treatments (T2 and T3) increased (*P* < 0.05) the nutrient digestibility, digestible crude protein (DCP), and total digestible nutrient (TDN), with T2 (EM_1_-treated DPLS) producing better (*P* < 0.05) results compared with El-Mofeed-treated DPLS (T3) (Table 2). The improved nutrient digestibility with additives-treated DPLS may be due to reduced fibre fraction, particularly ADL, of the diets. Lignin is a recalcitrant fibre that resists both microbial and enzymatic digestion and consequently lowers nutrient digestibility (Olafadehan and Adebayo 2016). Moreover, the additive treatments must have promoted faster degradation and clearance from the gastrointestinal tract, in tandem with the previous submissions of Olafadehan (2013). The improved fibre digestibility of the additive treatments suggests improved ruminal fermentation efficiency and microbial biomass production, increased number and/or activity of ruminal microbiota, and enhanced colonization and attachment of the ruminal microbes to the digesta (Nsereko et al. 2000; Wang et al. 2001; Olafadehan 2013). The chemical composition, TDN, and DCP of a diet have been used as indices of the nutritive value (Olafadehan 2013). Both TDN and DCP are products of apparent digestibility and dietary nutrient profile. Therefore, the enhanced chemical composition and nutrient digestibility of the additive treatments are responsible for the improved TDN and DCP. Based on the chemical composition (higher CP and lower fibre fraction), EM_1_ produced silage of superior quality relative to El-Mofeed produced silage. This is reflected in the greater nutrient digestibility, DCP, and TDN of the EM_1_ treatment relative to El-Mofeed treatment. These results suggest EM_1_ as a better additive for ensiling of crop residues than El-Mofeed. EM_1_ additive is a combination of desirable fermentative organisms or inoculants such as lactic acid bacteria, yeasts, and fermenting fungi, while El-Mofeed is a kind of additive containing fermentation/ensiling aiding nutrients such as molasses and urea. Abd-El-Ghany et al. (2016) earlier showed EM_1_-treated berseem hay improved nutrient digestion and nutritive value of the hay in rabbit diet. Kholif et al. (2005) and Mahrous et al. (2005) found that fungal treated roughages increased (*P* < 0.05) nutrient digestibility and nutritive value (TDN and DCP).

**Table 2:** Apparent digestibility and nutritive value of additives-treated date palm leaves silage fed to lambs

Item (%)	Experimental treatments			± SEM
	T1	T2	T3	
Dry matter	54.12^c^	64.77^a^	60.18^b^	0.18
Organic matter	54.53^c^	63.23^a^	60.47^b^	0.22
Crude protein	55.34^c^	63.3^a^	60.94^b^	0.50
Ether extract	68.2^c^	75.74^a^	74.2^b^	0.34
Non-fibre carbohydrate	58.68^c^	65.1^a^	62.4^b^	0.05
Neutral detergent fibre	58.88^c^	64.40^a^	62.60^b^	0.32
Acid detergent fibre	41.33^c^	55.43^a^	54.50^b^	0.45
Acid detergent lignin	32.62^c^	38.82^a^	39.85^b^	0.65
Cellulose	14.48^c^	19.95^a^	16.92^b^	0.22
Hemicellulose	47.83^c^	58.89^a^	54.32^b^	0.34
Nutritive value				
Total digestible nutrient	52.04^c^	60.59^a^	54.42^b^	0.55
Digestible crude protein	5.63^c^	7.32^a^	6.35^b^	0.05

^a, b, c^Means in the same row with different superscript are significantly (*P* < 0.05)

### Rumen parameters

Ruminal pH followed this rank order: T1 < T3 < T2 (*P* < 0.05) (Table 3). Greater ruminal pH of the treatments may be due to the lower fibre fractions of the diets. Low fibre diets reduced rates of chewing and saliva secretion and flow into the rumen to buffer the ruminal pH (Olafadehan and Adebayo 2016; Olafadehan et al. 2016; Okunade and Olafadehan 2019). Sheep fed untreated DPLS (control) had highest (*P* < 0.05) ruminal NH_3_-N values at 0 and 4 h post feeding followed by T2 and T3. The ruminal NH_3_-N concentrations for all treatments at 0 and 4 h post feeding were greater than the minimal level of 5 g NH_3_-N/L required for optimum rumen microbes growth and activity (Satter and Slyter 1974). This may be related to improved utilization of the dietary energy and enhanced fermentation in the rumen. Molina-Alcaide and Nefzaoui (1996) indicated that feeding date palm cake to sheep resulted in favourable pH (6.2 to 7.2) for fibrolytic activity. Higher ruminal NH_3_-N of T1 indicates rapid proteolysis of the dietary protein, in consonance with earlier reports (Olafadehan and Adebayo 2016; Olafadehan et al. 2020). Excess dietary N loss via urine due to rapid proteolysis contributes to environmental degradation and inefficiencies in ruminant production (Olafadehan et al. 2020). Lower ruminal NH_3_-N of the treatments, particularly T3, is beneficial as it indicates reduced dietary CP deamination and loss. Hassan et al. (2005) reported increased ruminal NH_3_-N for rams fed untreated banana wastes compared to biologically treated groups. Treatments T2 and T3 significantly (*P* < 0.05) increased the concentration of VFA. Increased VFA for the treatments may be due to improved nutrient digestibility and ruminal fermentation (Kholif et al. 2018a, b). The results are in agreement with those obtained by Mahrous et al. (2011) and Bassuny et al. (2003) who reported that rumen VFA concentrations were significantly increased with biological and chemical treatments of roughages. The decreased ruminal NH_3_-N and enhanced total VFA concentrations of treatments relative to the control are nutritionally desirable for improved performance of ruminants because VFA is used as an energy source and as substrates for the synthesis of other compounds (Kholif et al. 2018a). Though both T2 and T3 enhanced ruminal fermentation efficiency relative to T1, T2 provided better results than T3.

**Table 3:** Rumen fermentation parameters of lambs fed additives-treated date palm leaves silage

Item	Experimental treatments				± SEM
	Time (h)	T1	T2	T3	
pH	0	6.44^c^	6.67^a^	6.52^b^	0.06
	4	6.40^a^	6.19^c^	6.20^b^	0.04
NH_3_-N (mg/100 ml)	0	14.05^a^	19.28^c^	16.51^b^	0.08
	4	20.95^c^	28.01^a^	24.39^b^	0.45
TVFA (meq/100 ml)*	0	9.54^c^	13.44^a^	12.50^b^	0.55
	4	12.62^c^	17.70^a^	14.68^b^	0.54

^a, b, c^Means within the same row with different superscripts differ (*P* < 0.05)

*TVFA total volatile fatty acids

### Blood serum parameters

In the present study, all the serum metabolites were within the normal physiological ranges (Merck 1991). Total serum protein, albumin, and globulin followed the rank order: T2 > T3 > T1 (*P* < 0.05) (Table 4). Increased serum total protein, albumin, and globulin with additives-treated DPLS diets may be attributed to improved metabolic process as a response to increased nutrient intake and digestibility, especially CP and OM as well as increased flow of microbial protein from the rumen (Yang et al. 1999). Moreover, Kumar et al. (1980) and Ayling (2014) reported that blood total proteins concentrations reflect the nutritional status of an animal and are positively correlated with dietary protein level. These results agree with those reported by Abd El-Razik et al. (2012) who found that lambs fed diets containing biologically treated rice straw had higher values of blood total protein, albumin, and albumin:globulin ratio. Blood serum creatinine, urea, AST, ALT, cholesterol, and glucose were not affected by treatments. Normal values and lack of treatment effect on serum creatinine, urea, AST, and ALT suggest normal liver activity and function (Olafadehan 2011a).

**Table 4:** Blood metabolites of lambs fed additives-treated date palm leaves silage

Item	Experimental treatments			± SEM
	T1	T2	T3	
Total protein (g/dl)	6.21^c^	6.98^a^	6.50^b^	0.35
Albumin (g/dl)	3.49^c^	3.79^a^	3.64^b^	0.19
Globulin (g/dl)	2.72^c^	3.19^a^	2.86^b^	0.26
Creatinine (mg/dl)	1.30	1.28	1.28	0.11
Urea nitrogen (mg/dl)	42.22	41.05	40.21	3.48
Alanine transaminase (U/ml)	19.50	20.44	19.49	2.61
Aspartate transaminase (U/ml)	39	41	40	3.67
Cholesterol (mg/dl)	150.2	152.1	149.9	4.40

^a, b, c^Means within the same row with different superscripts differ (*P* < 0.05)

### Growth performance and economic efficiency

Total body gain, daily gain, and feed intake were higher (*P* < 0.05) for lambs fed additives-treated DPLS diets compared with control, but T2 improved these parameters relative to T3. Feed conversion was lowest for T2 followed by T3 and T1 (Table 5). Higher feed intake of additives treatments is due to improved palatability, reduced fibre, and greater CP and digestibility of the diets, in concurrence with previous reports (Olafadehan et al. 2014) who attributed improved intake in goats fed bovine rumen content to increased palatability, acceptance and consumption, rumen fermentation and digestibility, and shortened transit time of the digesta passage through the gastrointestinal tract. The greater intake of T2 than T3 further illustrates the superior nutritive value of EM_1_-treated DPLS relative to the El-Mofeed-treated DPLS. Body weight gain in animals is largely a function of feed intake, ruminal fermentation efficiency, and digestibility. Therefore, the reduced weight gain of the control goats is obviously the fallout of the decreased intake, ruminal fermentation, and digestibility, while the increased weight gain of the treatments is the result of enhanced feed utilization. Using enzyme additive, Beauchemin et al. (1995) attributed improvement in live weight with enzyme additive to increased digestibility, which yields more energy and/or nutrient to rumen microbes. Treatments T2 and T3 increased daily gain by 32.1 and 24.3%, respectively, relative to the control, while daily gain was 6.3% greater in T2 compared to T3. Improved feed utilization of T2 and T3 resulted in reduced feed conversion relative to T1. Similar trend was obtained by El-Marakby (2003) and Mahrous et al. (2005) who reported that groups fed ration containing biologically treated wheat straw or cotton stalks had improved feed conversion relative to the group fed the untreated roughages. Also, El-Marakby (2003) indicated that groups fed biologically treated roughages (cotton stalks or wheat straw) had better efficient feed conversion. Though total daily feed cost was lower (*P* < 0.05) in T1, price of daily gain, daily profit, and economics of feed efficiency increased in T2 and T3 compared to T1, with T2 having greater values than T3 (Table 5). Feed cost/kg LW gain was reduced (*P* < 0.05) in T2 and T3, indicating that it was significantly cheaper and more economical to produce 1 kg of LW with additives-treated DPLS than for the control untreated DPLS. This is in agreement with the previous studies where processed cassava peels reduced feed cost/kg LW gain relative to the unprocessed peels in rabbit diets (Olafadehan 2011b). There were 18.6 and 16.1% reductions in feed cost/kg LW gain in T2 and T3, respectively, relative to the T1. Daily profit and economics of feed efficiency were 65.5 and 44.8% and 52.6 and 38.1% greater for T2 and T3, respectively, relative to T1. El-Tahan et al. (2013) observed improved net revenue and economic efficiency in sheep fed fungi-treated palm fronds. Abd-El-Ghany et al. (2016) reported increased economic efficiency in rabbits fed diets containing EM_1_-treated conocarpus at 15 or 30% compared to those fed the control diet. Relative daily profit was 14.2% higher in T2 than in T3, indicating superior nutritive and economic benefit of EM_1_-treated DPLS relative to El-Mofeed-treated DPLS in the lambs’ diets. Olafadehan et al. (2018) attributed greater economic benefits in goats fed *Piliostigma thonningii* foliage in partial replacement of concentrate to superior quality of the foliage relative to the locally produced concentrate.

**Table 5:** Intake, growth, and economics of production of lambs fed additives-treated palm date leaves silage

Item	Experimental treatments			± SEM
	T1	T2	T3	
Initial live body weight, Kg	19.60	19.40	19.40	0.02
Final live body weight, Kg	36.50^c^	41.80^a^	40.40^b^	1.21
Total body gain, Kg	16.90^c^	22.40^a^	21.00^b^	1.23
Daily gain, g	140.80^c^	186.60^a^	175.00^b^	1.69
Feed intake/day (DMI) (g)				
CFM	650^c^	700^a^	680^b^	0.93
DPLS	200^c^	260^a^	240^b^	2.45
Total DM, g	850^c^	960^a^	920^b^	2.11
Feed conversion (DMI Kg/Kg gain)	6.03^a^	4.62^c^	5.25^b^	1.32
Economic efficiency				
Price of daily gain, LE	8.40^c^	11.19^a^	10.50^b^	1.02
Daily feed cost (LE)				
Cost of CFM consumed	2.93^c^	3.15^a^	3.06^b^	0.45
Cost of DPLS consumed	0.14^c^	0.18^a^	0.16^b^	0.03
Total daily feed cost, LE	3.07^c^	3.33^a^	3.22^b^	0.34
Feed cost/kg gain, LE	36.54^a^	29.75^c^	30.66^b^	0.43
Daily profit, LE	4.26^c^	7.05^a^	6.17^b^	1.66
Economics of feed efficiency,%*	138.76^c^	211.71^a^	191.61^b^	0.02
Relative feed cost, %**	–	81.41^b^	83.90^a^	0.23
Relative daily profit, %***	–	165.40^a^	144.83^b^	1.02

1 t of CFM = 4500 LE. 1 t of untreated DPL = 600 LE. 1 t of EM1-treated DPLS = 700 LE. 1 t of El-Mofeed-treated DPLS = 700. Market price of 1 kg live body weight = 60 LE

*Economic feed efficiency, % = daily profit/daily feed cost × 100

**Relative feed cost, % = feed cost, LE/kg gain (T2 and T3)/T1

***Relative daily profit, % = Daily profit LE (T2 and T3)/T1

^a, b, c^Means within the same row with different superscripts differ (*P* < 0.05)

## Conclusion

It can be concluded that incorporation of EM_1_- and El-Mofeed-treated DPLS in lambs’ total mixed rations improves voluntary intake, ruminal fermentation, nutrient digestibility, nutritive value, economic benefit, and growth performance. However, EM_1_ additive improved the quality of DPLS and performance of lambs compared with El-Mofeed additive.
